# Association between inhaled corticosteroids and upper respiratory tract infection in patients with chronic obstructive pulmonary disease: a meta-analysis of randomized controlled trials

**DOI:** 10.1186/s12890-020-01315-3

**Published:** 2020-10-28

**Authors:** Hong Chen, Yulin Feng, Ke Wang, Jing Yang, Yuejun Du

**Affiliations:** 1 Department of Infectious Disease, Chengdu Second People’s Hospital, No. 10 Qingyun South Street, Chengdu, 610017 China; 2 Department of Respiratory Medicine and Critical Care Medicine, West China Hospital, Sichuan University, No. 37 Guo Xue Xiang, Chengdu, 610041 China

**Keywords:** Inhaled corticosteroids (ICS), Chronic obstructive pulmonary disease (COPD), Upper respiratory tract infection (URTI), Risk, Meta-analysis

## Abstract

**Background:**

We aimed to assess the association between inhaled corticosteroids (ICSs) and the risk of upper respiratory tract infection (URTI) in patients with chronic obstructive pulmonary disease (COPD).

**Methods:**

PubMed, Embase, Cochrane Library and Clinical Trials.gov were searched from inception to October 2019. Randomized controlled trials (RCTs) of any ICSs vs control for COPD with reporting of URTI as an adverse event were included. The study was registered with PROSPERO prospectively (#CRD42020153134).

**Results:**

Seventeen RCTs (20,478 patients) were included. ICSs significantly increased the risk of URTI in COPD patients (RR, 1.13; 95% CI 1.03–1.24; *P* = 0.01; heterogeneity: *I*^*2*^ = 7%). Futher subgroup analyses suggested that short-term use of ICSs increased the risk of URTI (RR, 1.29; 95% CI 1.06–1.56; *P* = 0.01; heterogeneity: *I*^*2*^ = 14%) but not for long-term use (RR, 1.08; 95% CI 0.97–1.2; *P* = 0.14; heterogeneity: *I*^*2*^ = 0%). Short-term use of high-dose fluticasone increased the risk of URTI (RR, 1.33; 95% CI 1.03–1.71; *P* = 0.03; heterogeneity: *I*^*2*^ = 0%) but not for long-term use (RR, 1.12; 95% CI 0.97–1.29; *P* = 0.13; heterogeneity: *I*^*2*^ = 50%). Medium-dose (RR, 0.97; 95% CI 0.71–1.32; *P* = 0.84; heterogeneity: *I*^*2*^ = 0%) and low-dose (RR, 1.39; 95% CI 0.92–2.1; *P* = 0.12; heterogeneity: *I*^*2*^ = 30%) fluticasone did not increase the risk of URTI regardless of duration. Neither mometasone (RR, 1.05; 95% CI 0.87–1.26; *P* = 0.61; heterogeneity: *I*^*2*^ = 0%) nor budesonide (RR, 1.08; 95% CI 0.77–1.5; *P* = 0.67; heterogeneity: *I*^*2*^ = 46%) increased the risk of URTI, regardless of dosage or duration.

**Conclusions:**

Long-term use of ICSs does not increase the risk of URTI in patients with COPD. Short-term use of high-dose fluticasone increases the risk of URTI in patients with COPD, but not mometasone or budesonide.

**Supplementary information:**

**Supplementary information** accompanies this paper at 10.1186/s12890-020-01315-3.

## Introduction

Chronic obstructive pulmonary disease (COPD) is currently the third leading cause of death and disability worldwide [1–3]. Exacerbation is the major reason for hospital admission of patients with COPD and related to a significantly worse survival outcome [4–6]. Inhaled corticosteroids (ICSs) or combined with long-acting bronchodilators have been recommended to treat COPD patients with repeated exacerbations [1].

Although ICSs are generally considered to be relatively safe and well tolerated in patients, some adverse effects associated with ICSs have also been observed, such as the development of oropharyngeal candidiasis [6], adrenal suppression [7], diabetes [8], and pneumonia [9]. However, the association between ICSs and risk of upper respiratory tract infection (URTI) remains unclear, though URTI is the most common respiratory infection and also an important cause of exacerbation of COPD [10]. The large prospective study Toward a Revolution in COPD Health (TORCH) trial reported ICSs might increase the morbidity of URTI in COPD patients [11]. Moreover, other randomized controlled trials (RCTs) reported different or even contrary outcomes, and most of these studies were inadequate to detect significant difference between ICSs treatment groups and control groups [12–17].

Whether ICSs increase the risk of URTI in COPD patients may depend on duration, dosage and type of ICSs. Lack of safety evidence may result in insufficient use or over use of ICSs. Therefore, we conducted this meta-analysis of RCTs to assess the association between ICSs use and the risk of URTI in patients with COPD. We also aimed to clarify the contributions of medication details for the association, including duration, dosage level and type of ICSs.

## Methods

### Study protocol

This meta-analysis was conducted in accordance with the Preferred Reporting Items for Systematic Reviews and Meta-Analyses (PRISMA) recommendations [18]. And the study was registered with PROSPERO prospectively (#CRD42020153134).

### Search strategy

Two reviewers independently searched PubMed, Embase, Cochrane Library and Clinical Trials.gov for eligible articles from inception to May 10, 2019, and updated on October, 16, 2019. Both free words and MeSH terms referring to inhaled corticosteroid and the risk of URTI were used as search terms, including “Pulmonary Disease, Chronic Obstructive” OR “chronic obstructive pulmonary disease” OR “COPD” OR “airflow obstruction, chronic” OR “chronic airflow obstruction” OR “chronic obstructive airway disease” OR “chronic obstructive lung disease” OR “Bronchitis” OR “emphysema” AND “ICS” OR “inhaled corticosteroids” OR “fluticasone” OR “flunisolide” OR “budesonide” OR “beclomethasone” OR “triamcinolone” OR “mometasone” OR “ciclesonide”. We also conducted a manual search using the reference lists of key articles.

### Eligibility criteria

Eligible studies were identified through the PICOS criteria (participants, interventions, comparators, outcomes and study design) [18]. Inclusion criteria included: (1) patients with COPD; (2) The interventions included any type of inhaled corticosteroids, including ICSs alone or combined with long-acting bronchodilators; (3) non-ICSs treatment as control, including placebo or other inhaled drugs of corticosteroid free; (4) only trials reporting data on URTI as the outcome were included; (5) Only RCTs were included. Exclusion criteria included: (1) non-RCTs, such as observational studies, case series and reviews; (2) non-English articles; (3) Patients with asthma or unknown diagnosis; (4) ICSs was used in both the treatment group and the control group.

### Data collection process

Two investigators independently extracted relevant data from the included RCTs into standardized collection forms for the outcomes and evidence. Disagreements between the two investigators were resolved by discussion, and a third investigator was consulted if necessary. The corresponding authors were contacted when relevant data were not available.

### Risk of bias assessment and quality of evidence

Two investigators independently performed the risk assessment using the Cochrane Collaboration risk of bias tool [19]. Any disagreements between the two investigators were resolved by discussion, and a third investigator was consulted if necessary. The included RCTs were assessed according to the following features (1) random sequence generation; (2) allocation concealment; (3) blinding of participants and personnel; (4) blinding of outcome assessment;(5) selective reporting; (6) incomplete outcome data; (7) other bias. Each item was assessed as low, unclear, or high risk of bias.

### Statistical analysis

We performed meta-analyses for quantitative data synthesis using Revman Software (v.5.3, Cochrane Collaboration, London, UK). The weights of each study were estimated by Mantel-Haenszel method. We calculated the risk ratio (RR) and 95% confidence interval (CI) for the risk of URTI. A two-tailed *p* value < 0.05 was set for statistical significance. Heterogeneity was assessed using the I^2^ test, with I^2^ > 50% indicating a substantial heterogeneity [20]. A random-effect model would be used when a substantial level of heterogeneity was found, otherwise a fixed-effect model would be used.

### Subgroup analysis

Subgroup analyses were conducted according to the following variables: (1) duration (long [≥ 6 months] and short [< 6 months]); (2) dosage of ICSs [21] (high dose [defined as > 500 μg/day of fluticasone propionate or equivalent], medium dose [defined as > 250–500 μg/day of fluticasone propionate or equivalent] and low dose [defined as 100–250 μg/day of fluticasone propionate or equivalent]); (3) type of ICSs, including fluticasone, mometasone, budesonide, and beclomethasone.

## Results

### Study selection and study characteristics

**Fig. 1:**
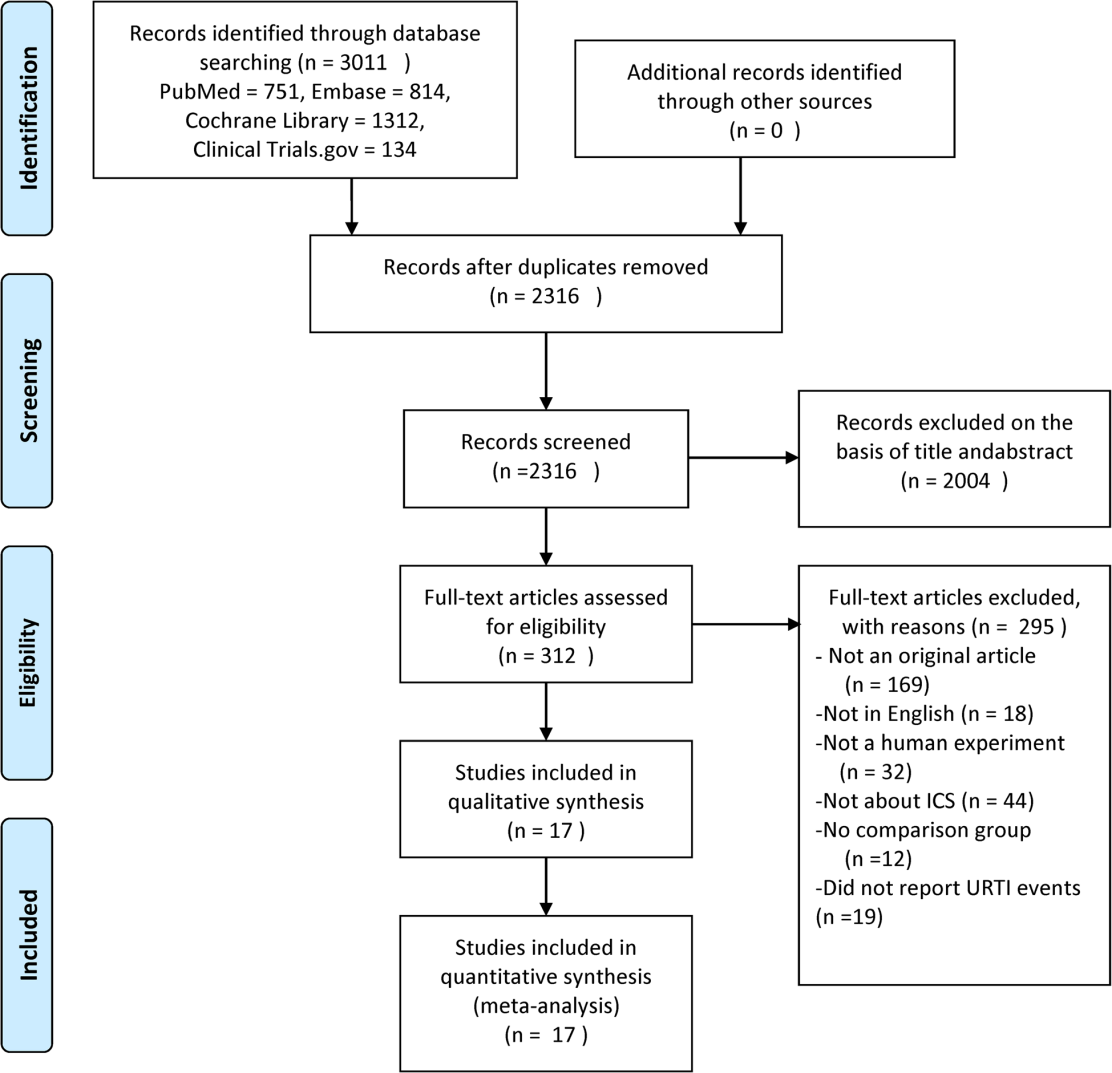
Study selection process

Figure 1 shows the study selection process. A total of 3011 references were identified after an initial search, and 17 RCTs [11–17, 22–31] including 20,478 patients were finally included in the meta-analysis. Of the 17 RCTs, 16 were multicenter, double-blind, randomized trials. These studies were published from 2002 to 2019, with population sizes ranging from 149 to 6184 participants. Duration of the trials ranged from 1 month to 36 months, with 10 trials [11, 12, 15, 16, 24–27, 29, 30] longer than or equal to 6 months, and 7 trials [13, 14, 17, 22, 23, 28, 31] shorter than 6 months. Eleven trials [11, 13, 15–17, 23, 25, 26, 28–30] investigated a high-dose ICSs treatment, 8 trials [12, 16, 22, 24, 27, 29–31] investigated a medium-dose ICSs treatment, and 5 trials [12, 14, 17, 27, 28] investigated a low-dose ICSs treatment. Fluticasone was evaluated in 10 trials [11, 13–15, 17, 22–24, 26, 28], mometasone in 4 trials [16, 25, 29, 30], budesonide in 3 trials [12, 27, 31], and beclomethasone in 1 trial [27]. Table 1 shows the main characteristics of the included studies.

**Table 1:** Characteristics of the 17 RCTs included in the meta-analysis of ICSs and risk of URTI

Study	Duration (months)	Mean Age (years)	FEV1 (%predicted)	Male (%)	Interventions	NO. of URTI/Total
Mahler et al. [13]	5.5	63.5	40.8	66.0	T:FP 500 μg bid or FSC (S 50 μg /FP 500 μg) bid	T:84/342
					C: Placebo or S 50 μg bid	C: 56/349
Hanania et al. [22]	5.5	63.8	41.8	63.2	T:FP 250 μg bid or FSC (S 50 μg /FP 250 μg) bid	T:40/361
					C: Placebo or S 50 μg bid	C: 42/362
Calverley et al. [11]	36	65.0	44.0	75.5	T:FP 500 μg bid or FSC (S 50 μg /FP 500 μg) bid	T:309/3098
					C: Placebo or S 50 μg bid	C: 277/3086
Zheng et al. [23]	5.5	66.2	47.0	89.2	T:FSC (S 50 μg /FP 500 μg) bid	T:32/297
					C: Placebo	C: 14/148
Ferguson et al. [24]	12	65.0	32.8	55.0	T:FP 250 μg bid	T:31/394
					C: S 50 μg bid	C: 31/388
Calverley et al. [25]	12	65.1	NR	68.3	T: MF 800 μg qd or MF 400 μg bid	T:164/616
					C: Placebo	C: 71/295
Anzueto et al. [26]	12	65.4	34	54.0	T:FSC (S 50 μg /FP 500 μg) bid	T:41/394
					C: Placebo	C: 30/403
Calverley et al. [27]	11	63.6	42.2	80.7	T:BDP/FM 200/12 μg bid or BUD/FM 400/12 μg bid	T:3/479
					C: FM 12 μg bid	C: 5/239
Tashkin et al. [30]	6	59.8	NR	77.5	T: MF/FM 200/10 μg bid or MF/FM 400/10 μg bid or MF 400 μg bid	T:19/634
					C: Placebo or FM 10 μg bid	C:13/421
Tashkin et al. [16]	12	59.7	39.1	76.0	T: MF/FM 200/10 μg bid or MF/FM 400/10 μg bid or MF 400 μg bid	T:58/1351
					C: Placebo or FM 10 μg bid	C: 39/900
Doherty et al. [29]	6	59.6	38.6	75.2	T: MF/FM 200/10 μg bid or MF/FM 400/10 μg bid or MF 400 μg bid	T:39/717
					C: Placebo or FM 10 μg bid	C: 26/479
Sharafkhaneh et al. [12]	12	63.0	37.7	62.0	T:BUD/FM 320/9 μg bid or BUD/FM 160/9 μg bid	T:90/815
					C: FM 9 μg bid	C: 39/403
Boscia et al. [28]	1	57.9	49.8	46.3	T:FF/VI 50/25 μg qd or FF/VI 100/25 μg qd or FF/VI 200/25 μg qd	T:2/98
					C: Placebo	C: 1/51
Vogelmeier et al. [15]	6	63.3	60.3	70.9	T: FSC (S 50 μg /FP 500 μg) bid	T:2/264
					C: QVA149 110/50 μg qd	C: 7/258
Kerwin et al. [14]	5.5	62.5	48.3	66.6	T:FF/VI 50/25 μg qd or FF/VI 100/25 μg qd or FF 100 μg qd	T:50/618
					C: Placebo or VI 25 μg qd	C: 19/412
Martinez et al. [17]	5.5	61.5	47.9	72.3	T:FF/VI 100/25 μg qd or FF/VI 200/25 μg qd or FF 100 μg qd or FF 200 μg qd	T:23/816
					C: Placebo or VI 25 μg qd	C: 14/408
Huang et al. [31]	2.8	64.1	NR	86.4	T: BUD/FM 320/9 μg bid, I 40 μg qid and T 100 mg bid	T:4/293
					C:I 40 μg qid and Th 100 mg bid	C: 2/289

*Abbreviations*: *ICSs* Inhaled corticosteroids, *URTI* Upper respiratory tract infection, *FEV*_*1*_ Forced expiratory volume in 1 s, *T* Treatment group, *C* Control group, *NR* Not reported, *FP* Fluticasone propionate, *S* Salmeterol, *FSC* Fluticasone propionate/salmeterol, *P* Placebo, *MF* Mometasone furoate, *BUD* Budesonide, *FM* Formoterol, *BDP* Beclomethasone, *QVA149* Indacaterol/glycopyrronium, *FF* Fluticasone furoate, *VI* Vilanterol, *I* Ipratropium, *Th* Theophylline, *QD* Once a day, *BID* Twice a day, *QID* Four times a day

### Risk of bias and quality of evidence

**Fig. 2:**
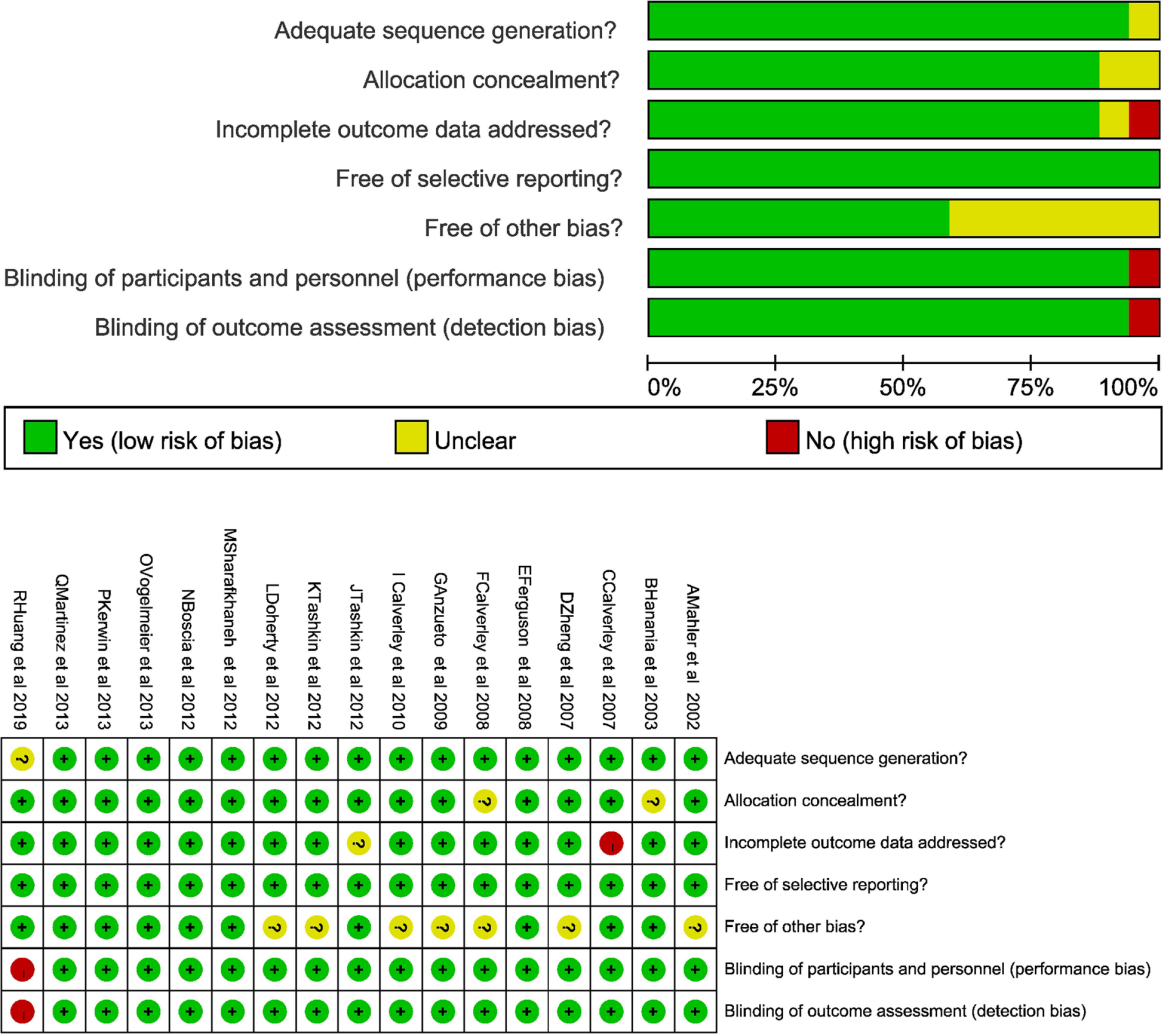
Risk of bias of the included studies

All trials were assessed using the Cochrane Collaboration risk of bias assessment tool. RCTs with four or more features were considered to be of high quality. Seventeen RCTs were regarded as high quality according to the risk of bias assessment tool and were included in the meta-analysis. All studies had low risk of selective reporting bias. One study had high risk of blinding of participants and personnel bias, 1 study had high risk of blinding of outcome assessment bias, and 1 study had high risk of incomplete outcome data. One study had unclear risk of random sequence generation bias, 2 studies had unclear risk of allocation concealment bias, 1 study had unclear risk of incomplete outcome data bias and 7 studies had other bias (Fig. 2).

### Risk of URTI associated with ICSs

**Fig. 3:**
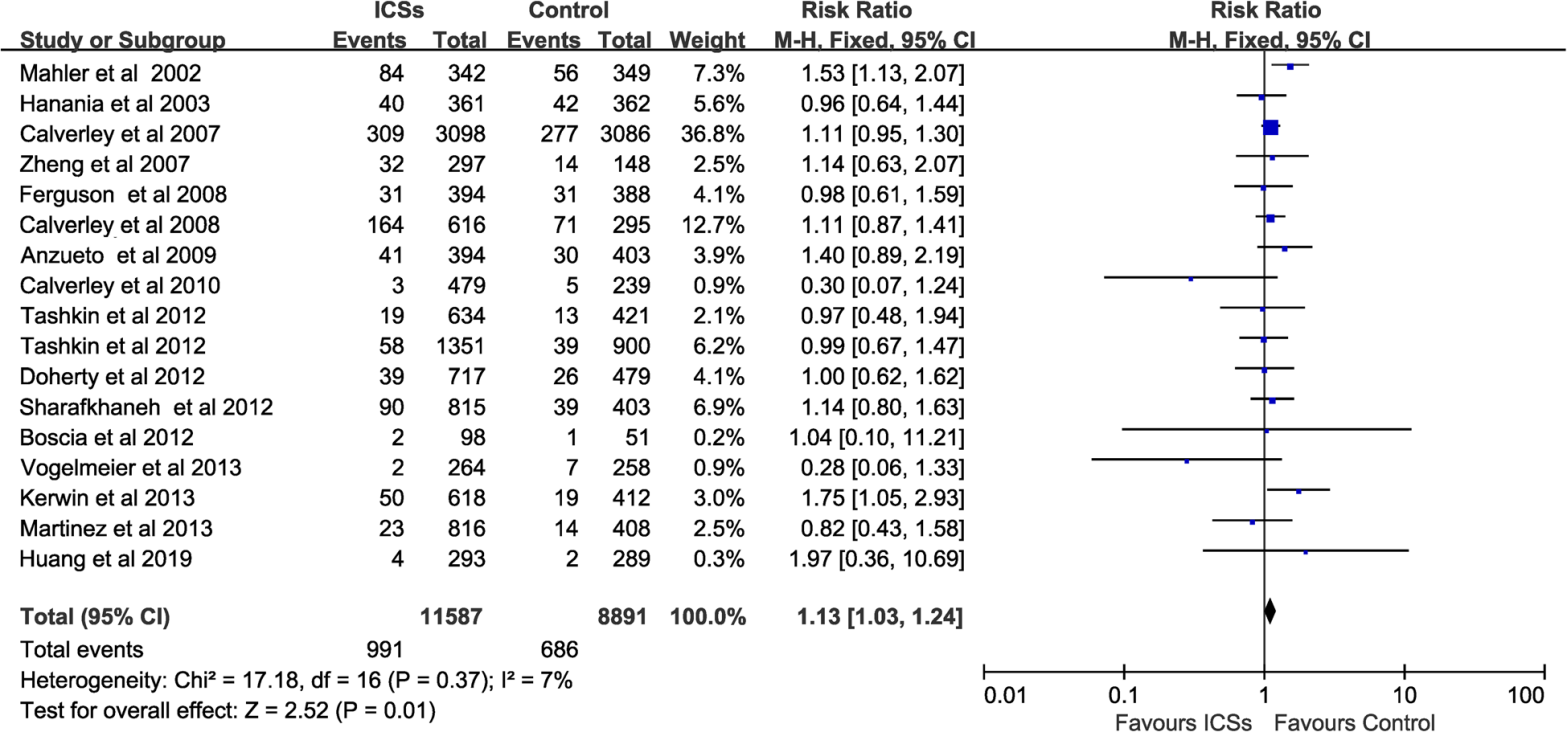
Risk of URTI associated with ICSs. URTI, upper respiratory tract infection; ICSs, inhaled corticosteroids

All 17 RCTs with 20,478 patients reported URTI as an adverse event. The risk of URTI was 8.6% (991 of 11,587 patients) in the ICSs treatment groups, 7.7% (686 of 8891 patients) in the control groups, and 8.2% in all patients (1677 of 20,478 patients). Compared with non-ICSs treatment, ICSs was associated with a significantly increased the risk of URTI in patients with COPD (RR, 1.13; 95% CI 1.03–1.24; *P* = 0.01; heterogeneity: *I*^*2*^ = 7%) (Fig. 3).

### Risk of URTI associated with ICSs for different durations

**Fig. 4:**
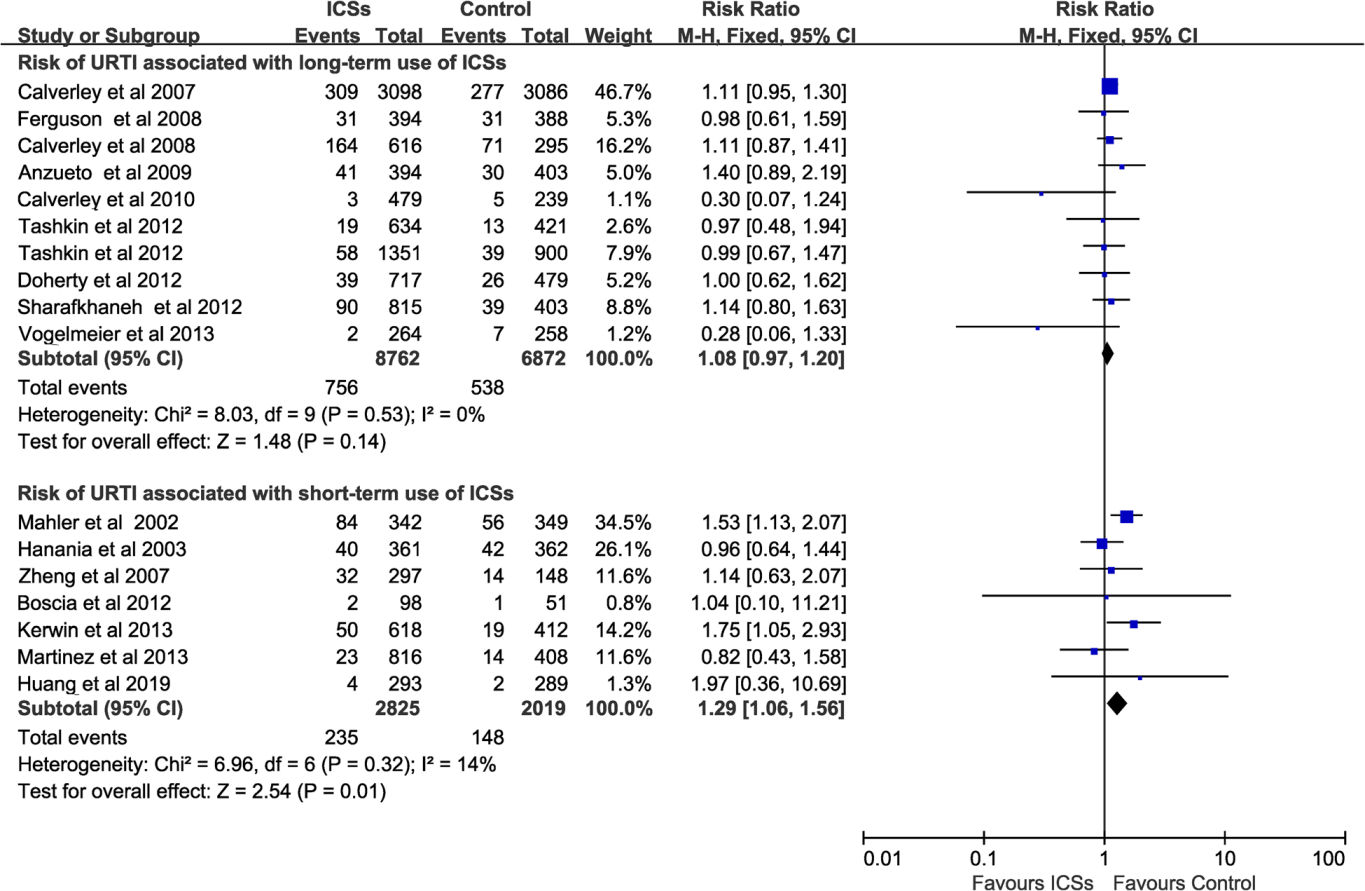
Risk of URTI associated with ICSs for different duration. URTI, upper respiratory tract infection; ICSs, inhaled corticosteroids

Long-term use of ICSs [11, 12, 15, 16, 24–27, 29, 30] (10 RCTs, 15,634 patients) did not increase the risk of URTI (RR, 1.08; 95% CI 0.97–1.2; *P* = 0.14; heterogeneity: *I*^*2*^ = 0%), whereas short- term use of ICSs [13, 14, 17, 22, 23, 28, 31] (7 RCTs, 4844 patients) was associated with a significantly increased the risk of URTI (RR, 1.29; 95% CI 1.06–1.56; *P* = 0.01; heterogeneity: *I*^*2*^ = 14%) (Fig. 4).

### Risk of URTI associated with different doses of ICSs

**Fig. 5:**
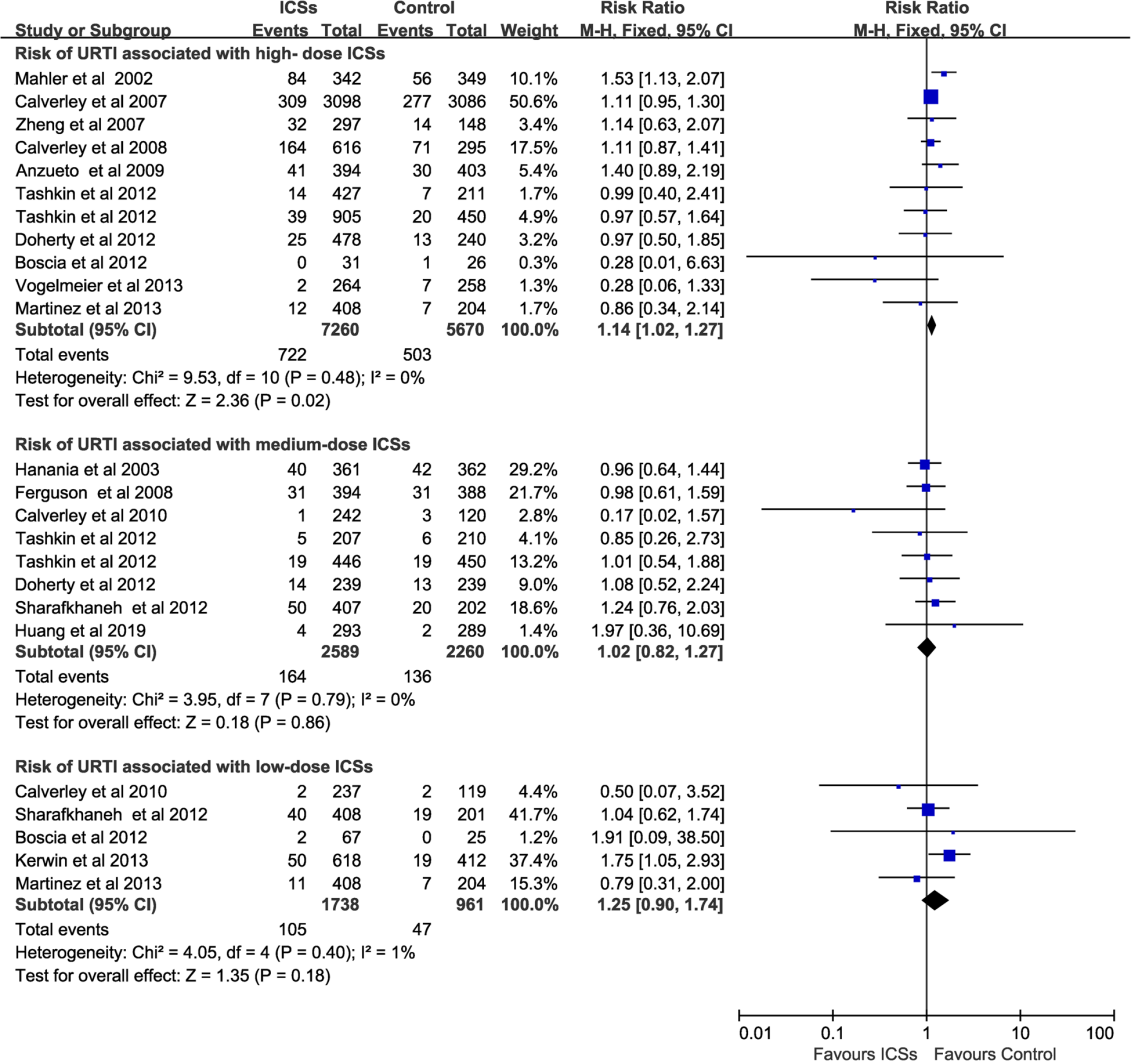
Risk of URTI associated with different doses of ICSs. URTI, upper respiratory tract infection; ICSs, inhaled corticosteroids

High-dose ICSs [11, 13, 15–17, 23, 25, 26, 28–30] treatment (11 RCTs, 12,930 patients) was associated with a significantly increased the risk of URTI (RR, 1.14; 95% CI 1.02–1.27; *P* = 0.02; heterogeneity: *I*^*2*^ = 0%), but neither medium-dose ICSs (8 RCTs, 4849 patients) [12, 16, 22, 24, 27, 29–31] (RR, 1.02; 95% CI 0.82–1.27; *P* = 0.86; heterogeneity: *I*^*2*^ = 0%) nor low-dose ICSs (5 RCTs, 2699 patients) [12, 14, 17, 27, 28] (RR, 1.25; 95% CI 0.9–1.74; *P* = 0.18; heterogeneity: *I*^*2*^ = 1%) increased the risk of URTI (Fig. 5).

### Risk of URTI associated with high-dose ICSs for different durations

**Fig. 6:**
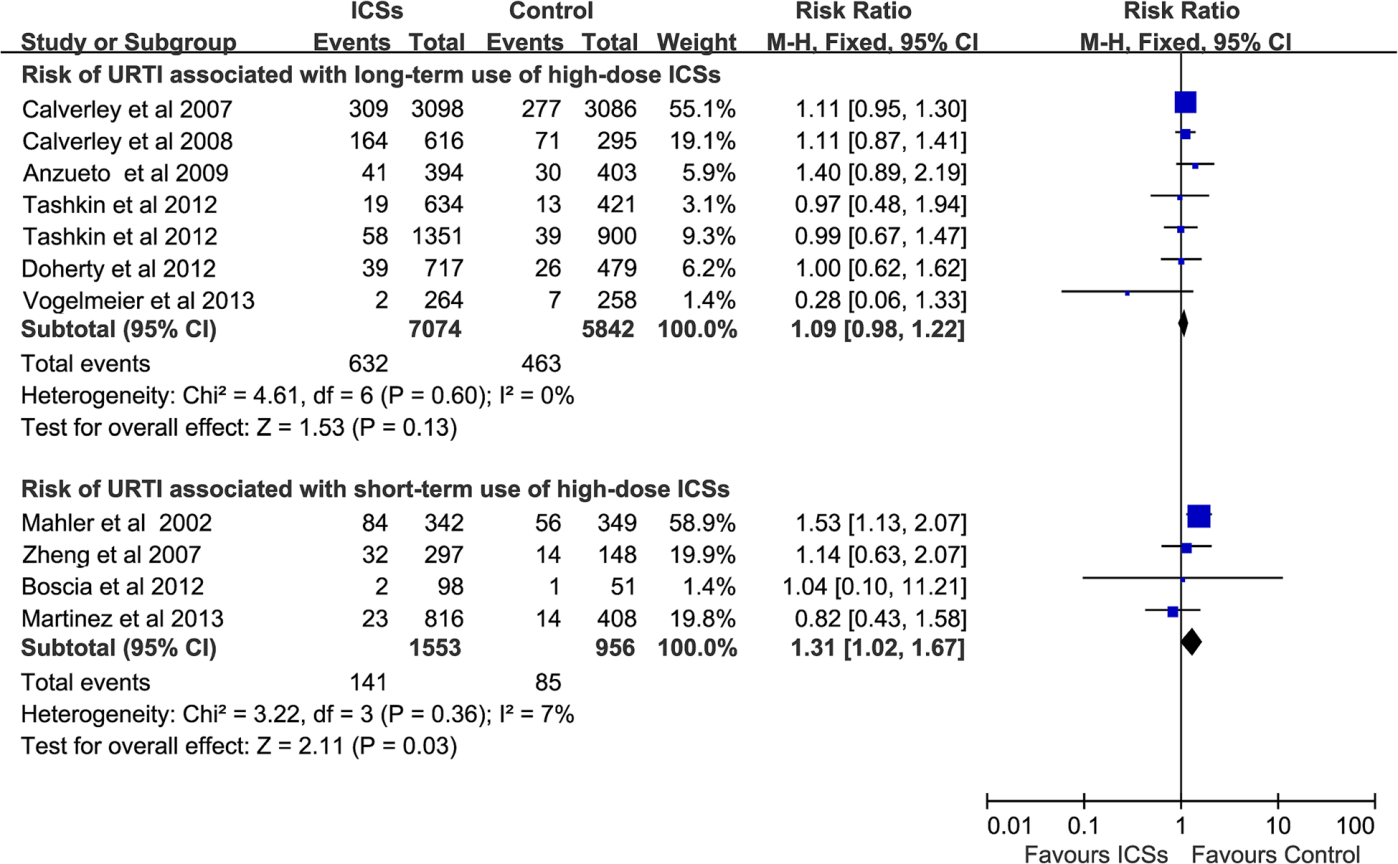
Risk of URTI associated with high-dose ICSs for different durations. URTI, upper respiratory tract infection; ICSs, inhaled corticosteroids

Long-term use of high-dose ICSs [11, 15, 16, 25, 26, 29, 30] (7 RCTs, 12,916 patients) did not increase the risk of URTI (RR, 1.09; 95% CI 0.98–1.22; *P* = 0.13; heterogeneity: *I*^*2*^ = 0%), whereas short- term use of high-dose ICSs [13, 17, 23, 28] (4 RCTs, 2509 patients) was associated with a significantly increased the risk of URTI (RR, 1.31; 95% CI 1.02–1.67; *P* = 0.03; heterogeneity: *I*^*2*^ = 7%) (Fig. 6).

### Risk of URTI associated with fluticasone

**Fig. 7:**
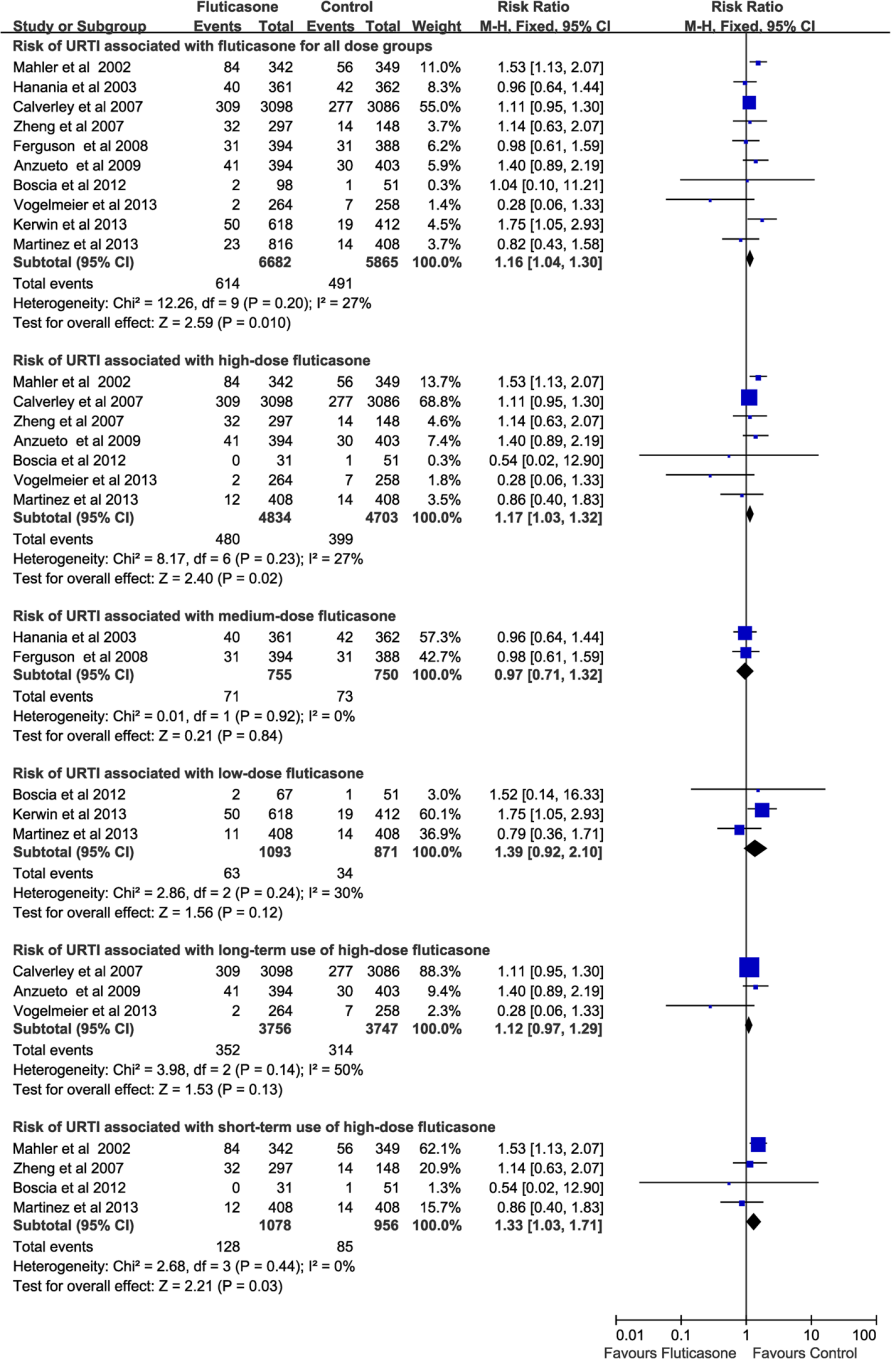
Risk of URTI associated with fluticasone. URTI, upper respiratory tract infection

Fluticasone [11, 13–15, 17, 22–24, 26, 28] (10 RCTs, 12,547 patients) was associated with a significantly increased the risk of URTI for all dose groups (RR, 1.16; 95% CI 1.04–1.3; *P* = 0.01; heterogeneity: *I*^*2*^ = 27%). Subgroup analyses suggested that high-dose fluticasone [11, 13, 15, 17, 23, 26, 28] (7 RCTs, 9537patients) was associated with a significantly increased the risk of URTI (RR, 1.17; 95% CI 1.03–1.32; *P* = 0.02; heterogeneity: *I*^*2*^ = 27%), but neither medium-dose fluticasone (2 RCTs, 1505patients) [22, 24] (RR, 0.97; 95% CI 0.71–1.32; *P* = 0.84; heterogeneity: *I*^*2*^ = 0%) nor low- dose fluticasone (3 RCTs, 1964 patients) [14, 17, 28] (RR, 1.39; 95% CI 0.92–2.1; *P* = 0.12; heterogeneity: *I*^*2*^ = 30%) increased the risk of URTI. Moreover, long-term use of high-dose fluticasone (3 RCTs, 7503 patients) [11, 15, 26] did not increase the risk of URTI (RR, 1.12; 95% CI 0.97–1.29; *P* = 0.13; heterogeneity: *I*^*2*^ = 50%), whereas short-term use of high-dose fluticasone (4 RCTs, 2034 patients) was associated with a significantly increased the risk of URTI (RR, 1.33; 95% CI 1.03–1.71; *P* = 0.03; heterogeneity: *I*^*2*^ = 0%) (Fig. 7).

### Risk of URTI associated with mometasone

**Fig. 8:**
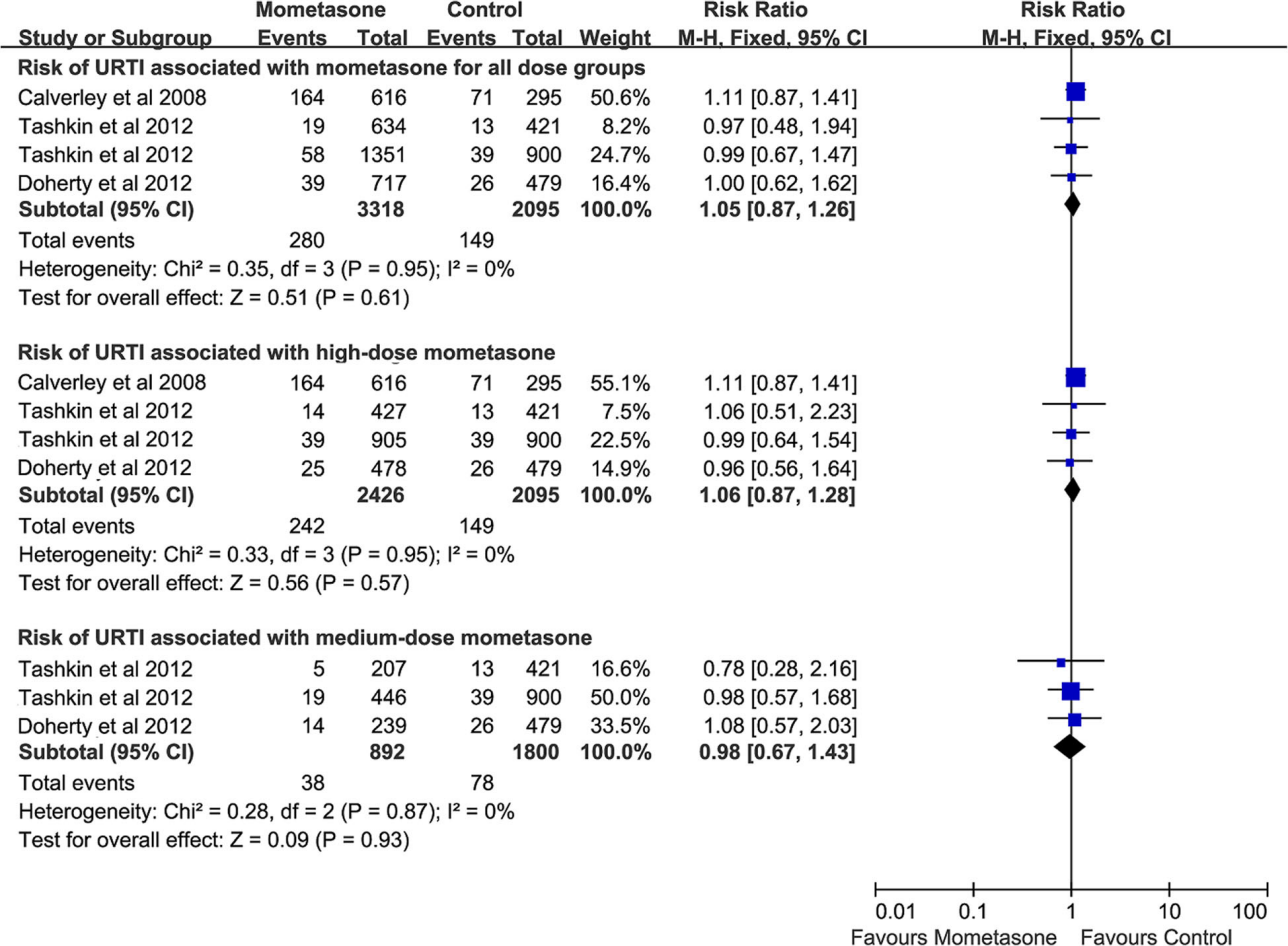
Risk of URTI associated with mometasone. URTI, upper respiratory tract infection

Mometasone [16, 25, 29, 30] (4 RCTs, 5413 patients) did not increase the risk of URTI for all dose groups (RR, 1.05; 95% CI 0.87–1.26; *P* = 0.61; heterogeneity: *I*^*2*^ = 0%). Subgroup analyses suggested that neither high-dose mometasone (4 RCTs, 4521 patients) [16, 25, 29, 30] (RR, 1.06; 95% CI 0.87–1.28; *P* = 0.57; heterogeneity: *I*^*2*^ = 0%) nor medium-dose mometasone (3 RCTs, 2692 patients) [16, 29, 30] (RR, 0.98; 95% CI 0.67–1.43; *P* = 0.93; heterogeneity: *I*^*2*^ = 0%) increased the risk of URTI (Fig. 8).

### Risk of URTI associated with budesonide

**Fig. 9:**
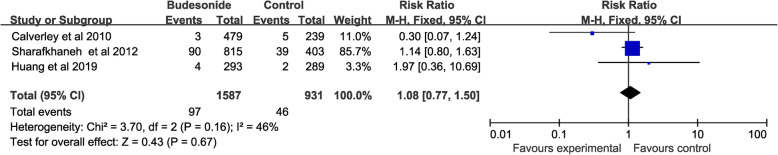
Risk of URTI associated with budesonide. URTI, upper respiratory tract infection

Budesonide [12, 27, 31] (3 RCTs, 2518 patients) did not increase the risk of URTI (RR, 1.08; 95% CI 0.77–1.5; *P* = 0.67; heterogeneity: *I*^*2*^ = 46%) (Fig. 9).

## Discussion

This meta-analysis of 17 multicenter RCTs (including 20,478 patients) suggested that short-term use of ICSs increased the risk of URTI but not for long-term use. Futher subgroup analyses revealed that only short-term use of high-dose fluticasone increased the risk of URTI but not for long-term use of high-dose fluticasone. Medium-dose and low-dose fluticasone did not increase the risk of URTI regardless of duration. Neither mometasone nor budesonide increased the risk of URTI, regardless of dosage or duration.

Exacerbation is common in patients with COPD affecting about 20% of patients with 40–45% of predicted FEV_1_ (1.3 events per year). Repeated exacerbations lead to worse survival outcome of patients [5]. Daily use of ICSs has been proved to decrease the frequency of exacerbations and improve quality of life in patients with FEV_1_ less than 50% predicted [1, 4]. However, daily use of ICSs may cause drug-related adverse events, such as increased risks of fracture and infections [32, 33], but may not increase the risk of cardiovascular events [34]. URTI is the most common respiratory infections and also an important cause of exacerbation of COPD. Moreover, URTI can significantly reduce the quality of life in patients [10]. However, the association between ICSs and the risk of URTI remains unclear.

Our results suggested that use of ICSs was associated with a significantly increased the risk of URTI in COPD patients. However, further subgroup analyses suggested that only short-term use of ICSs significantly increased the risk of URTI but not for long-term use of ICSs in COPD patients. The result was unexpected. We speculated that short-term use of ICSs could not effectively control the airway inflammation, whereas the immunosuppression effects of corticosteroids due to local high concentration might cause susceptibility to URTI in patients with COPD. On the contrary, long-term use of ICSs may improve health status of COPD patients by decreasing the frequency of exacerbations and improving clinical symptoms. In addition, the immune compensation mechanism of patients may play a role in resisting the immunosuppression of corticosteroid in the long term. A previous RCT conducted by Eichenhorn et al. may support our findings [35]. In their study, Eichenhorn et al. evaluated the effects of inhaled triamcinolone on adrenal function in 221 patients who were recruited from patients already enrolled in the Lung Health Study II, and found that use of triamcinolone (1200 μg daily) for 3 years did not have suppression effects on adrenal function [35, 36].

Subgroup analyses were performed according to different dosage of ICSs. The results suggested that high-dose ICSs significantly increased the risk of URTI but not for medium- and low-dose ICSs. These findings may be explained by the possible dose-response effect of ICSs [7, 37]. In the further analysis, we found that only short-term use of high-dose ICSs was associated with a significantly increased the risk of URTI but not for long-term use of high-dose ICSs. The results further supported that long-term use of ICSs did not increase ICS-related risk of URTI.

We also conducted subgroup analyses according to type of ICSs. The results suggested that high-dose fluticasone was associated with a significantly increased risk of URTI but not for mometasone, budesonide, and medium- and low-dose fluticasone. Previous studies have reported that fluticasone may effectively suppress innate immunoresponse of alveolar macrophages, and the immunosuppressive effects of fluticasone might be tenfold greater than those of budesonide in human airways [38, 39]. Moreover, the included RCTs mainly explored medium- or low-dose budesonid rather than high-dose. Similarly, we also found that short-term use of high-dose fluticasone was associated with a significantly increased the risk of URTI but not for long-term use of high-dose fluticasone. The results were consistent with the above results of the merged different types of ICSs.

Our results were not consistent with a previous meta-analysis conducted by Yang et al. in 2016 [40], which first reported that long-term use of ICSs may increase the risk of URTI in patients with COPD. However, that meta-analysis had limitations mainly because it did not include several large multicenter RCTs (including 3507 patients) published recently [14, 15, 17, 28, 31]. The smaller sample size of the previous meta-analysis may weaken the reliability and generalizability of the conclusion. Moreover, the previous meta-analysis failed to provide implication for clinical practice due to lack of subgroup analyses according to medication details including duration, dosage level and type of ICSs.

The strengths of our study were that we used a comprehensive search strategy and explicit inclusion criteria including 17 multicenter RCTs (20,478 patients), which met the requirements of sequential analysis. In addition, we conducted multiple subgroup analyses according to medication details including duration, dosage, and type of ICSs, that minimized the heterogeneity of pooled analyses and provided implications for clinical practice.

This study also had some limitations which mainly stemed from the quality of reported data. First, since some adverse events (such as URTI) were not the predefined outcomes and there were no homogeneous definitions among the clinical trials, these adverse events may be misclassified. This inherent methodological defect of clinical trials is one of the factors limiting the results of meta-analyses on drug safety. However, as most of the included trials in this meta-analysis were double blind, such misclassification would not have a substantial impact on the results of the meta-analysis, because the direction of bias may be toward the null. Second, the possibility of asthma-COPD overlap (ACO) in the study population may be one of the confounding factor of results. However, as a result of randomization, this confounding factor could not substantially affect the results of the meta-analysis as ACO may be approximately evenly distributed between the ICSs treatment group and the control group. Third, further subgroup analyses could not be performed due to lack of some patient related information, such as eosinophil count, exacerbations in the past year, comorbidities, smoking status and preexisting ICSs use and this may be a confounding factor of results.

## Conclusions

Long-term use of ICSs does not increase the risk of URTI in patients with COPD. Short-term use of high-dose fluticasone increases the risk of URTI in patients with COPD, but not mometasone or budesonide.

## Supplementary information

**Additional file 1.** Search strategy.

## Data Availability

The datasets used and/or analysed during the current study are available from the corresponding author on reasonable request.

## Abbreviations

ICSs: Inhaled corticosteroids
URTI: Upper respiratory tract infection
COPD: Chronic obstructive pulmonary disease
RCTs: Randomized controlled trials
FEV_1_: Forced expiratory volume in 1 s
TORCH: The large prospective study Toward a Revolution in COPD Health
PRISMA: Preferred Reporting Items for Systematic Reviews and Meta-Analyses
