# Supplementary material for: Association between inhaled corticosteroids and upper respiratory tract infection in patients with chronic obstructive pulmonary disease: a meta-analysis of randomized controlled trials
Source: BMC Pulm Med. 2020 Oct 28;20:282. doi: 10.1186/s12890-020-01315-3 (PMC7594481; doi:10.1186/s12890-020-01315-3)
Supplement: Supplementary file 1 — Additional file 1. Search strategy. [file 12890_2020_1315_MOESM1_ESM.doc]

**Supplementary file:** Search strategy

| **Database** | **Search strategy** |
| --- | --- |
| Cochrane Library | #1 MeSH descriptor: [Pulmonary Disease, Chronic Obstructive] explode all trees |
|  | #2 (chronic obstructive pulmonary disease):ti,ab,kw |
|  | #3 (chronic airﬂow obstruction):ti,ab,kw |
|  | #4 (COPD):ti,ab,kw |
|  | #5 (chronic obstructive lung disease):ti,ab,kw |
|  | #6 (airﬂow obstruction, chronic):ti,ab,kw |
|  | #7 (chronic obstructive airway disease):ti,ab,kw |
|  | #8 (emphysema):ti,ab,kw |
|  | #9 (bronchitis):ti,ab,kw |
|  | #10 #1 OR #2 OR #3 OR #4 OR #5 OR #6 OR #7 OR #8 OR #9 |
|  | #11 (ICS):ti,ab,kw |
|  | #12 (inhaled corticosteroids):ti,ab,kw |
|  | #13 (fluticasone):ti,ab,kw (Word variations have been searched) |
|  | #14 (flunisolide):ti,ab,kw |
|  | #15 (budesonide):ti,ab,kw |
|  | #16 (beclomethasone):ti,ab,kw |
|  | #17 (mometasone):ti,ab,kw |
|  | #18 (triamcinolone):ti,ab,kw |
|  | #19 (ciclesonide):ti,ab,kw |
|  | #20 #11 OR #12 OR #13 OR #14 OR #15 OR #16 OR #17 OR #18 OR #19 |
|  | #21 #19 AND #20 |
| Embase | #1 'chronic obstructive lung disease'/exp |
|  | #2 'chronic obstructive pulmonary disease' OR copd OR 'chronic airﬂow obstruction' OR 'chronic obstructive airway disease' OR 'emphysema' OR 'bronchitis':ab,ti |
|  | #3 #1 OR #2 |
|  | #4 ics OR 'inhaled corticosteroids' OR 'fluticasone'/exp OR fluticasone OR 'flunisolide'/exp OR flunisolide OR 'budesonide'/exp OR budesonide OR 'beclomethasone'/exp OR beclomethasone OR 'mometasone'/exp OR mometasone OR 'triamcinolone'/exp OR triamcinolone OR ciclesonide:ab,ti |
|  | #5 #3 AND #4 AND [article]/lim AND [english]/lim |
|  | #6 #5 AND ('human'/de OR 'randomized controlled trial topic'/de) |
| PubMed | #1 Search: ((((((((ICS[Text Word]) OR (inhaled corticosteroids[Text Word])) OR (fluticasone[Text Word])) OR (flunisolide[Text Word])) OR (budesonide[Text Word])) OR (beclomethasone[Text Word])) OR (mometasone[Text Word])) OR (triamcinolone[Text Word])) OR (ciclesonide[Text Word]) |
|  | #2 Search: (((((((chronic obstructive pulmonary disease[Text Word]) OR (pulmonary disease, chronic obstructive[Text Word])) OR (COPD[Text Word])) OR (chronic airﬂow obstruction[Text Word])) OR (airﬂow obstruction, chronic[Text Word])) OR (chronic obstructive airway disease[Text Word])) OR (emphysema[Text Word])) OR (Bronchitis[Text Word]) |
|  | #3 Search: #1 AND #2 |
|  | #4 Search: #3 AND Filters: Humans |
|  | #5 Search: #4 AND Filters: Humans, English |
|  | #6 Search: #5 AND Filters: Controlled Clinical Trial, Humans, English |
|  | #7 Search: #5 AND Filters: Clinical Trial, Controlled Clinical Trial, Humans, English |
|  | #8 Search: #5 AND Filters: Clinical Trial, Controlled Clinical Trial, Randomized Controlled Trial, Humans, English |
| Clinical Trials.gov | Search term: (COPD OR chronic obstructive lung disease) AND ( ICS OR inhaled corticosteroids OR fluticasone OR flunisolide OR budesonide OR beclomethasone OR mometasone OR triamcinolone OR ciclesonide) |
|  | Study type: Intervention |
